# Management of choroidal/ciliary body metastasis in the era of targeted cancer therapy

**DOI:** 10.3389/fonc.2024.1516783

**Published:** 2024-12-20

**Authors:** Moses O. Evbuomwan, Rupak Bhuyan, Uwajachukwumma A. Uzomah, Farzad Jamshidi, Carryn Anderson, H. Culver Boldt, Elaine Binkley

**Affiliations:** ^1^ Department of Radiation Oncology, University of Iowa, Iowa City, IA, United States; ^2^ Department of Ophthalmology and Visual Sciences, University of Iowa, Iowa City, IA, United States; ^3^ Institute for Vision Research, University of Iowa, Iowa City, IA, United States

**Keywords:** choroid, metastasis, external beam radiation, chemotherapy, targeted biologic

## Abstract

**Introduction:**

Choroidal metastases from systemic malignancies are the most common intraocular malignancies in adults. External beam radiation (EBR) has historically been first-line therapy for metastatic tumors to the choroid. However, good responses have been described with newer targeted biologics. The optimal management strategy for patients with choroidal metastatic tumors in the era of targeted cancer therapy is not known. We aim to describe management of these tumors in a “real-world” setting using both radiation and systemic therapy.

**Methods:**

We conducted a retrospective review of patients with choroidal/ciliary body metastases managed by the ocular oncology service at our institution over a five-year period. Demographic data, tumor type, treatment, visual outcomes, and mortality data were recorded.

**Results:**

26 patients (33 eyes) with choroidal/ciliary body metastasis were identified. Primary malignancies included lung (8) breast (8), renal (3), esophageal (3), carcinoid (2), squamous cell carcinoma of the tonsil (1), and testicular cancer (1). Average time from diagnosis of ocular metastasis to death was 8 months (1-34). 20 eyes were treated with EBR and 13 eyes were treated with other modalities. Final logMAR visual acuity for eyes treated with radiation was 0.11 (0-3). Final visual acuity for eyes treated with other modalities was 0.18 (0-.70), with local tumor control in 20/23 eyes that had follow up after treatment. The difference between final visual acuity in these groups was not visually significant p=0.48.

**Conclusion:**

Patients with choroidal/ciliary body metastasis treated with either EBR or systemic therapy can have good visual outcomes. More work needs to be conducted to determine the optimal first-line treatment of ocular metastasis for specific tumor subtypes.

## Introduction

Choroidal metastases from systemic malignancies are the most common intraocular malignancies in adults. The most common tumor metastatic to the choroid in men is lung cancer, and breast cancer is most common in women (1). There are also many other primary tumor types that metastasize to the choroid and ciliary body (2–5). While management of these lesions is palliative, they can significantly affect patient quality of life as they cause vision loss in individuals battling end-stage cancer.

The gold standard for the management of choroidal metastasis has been external beam radiotherapy or plaque brachytherapy due to the poor response of many of these tumors to traditional systemic chemotherapy (6). Other modalities such as proton beam therapy, gamma knife radiation, transpupillary thermotherapy, photodynamic therapy, and adjunct anti-VEGF injections have also been used in select cases (7).

With the advent of targeted biologic therapies and immunotherapies over the last several years, good responses of choroidal tumors have been described with several of these agents (8–13). Given the treatment burden and risk for side effects such as radiation retinopathy, dry eye, and cataract progression with external beam radiation, determining which situations could be managed with systemic therapy versus radiation therapy is important. This is particularly relevant for patients with poor prognosis and limited survival time, and for patients with an anticipated longer survival time, in whom the risk for vision loss from radiation retinopathy is higher. Here we describe outcomes for consecutive patients with choroidal/ciliary body metastasis presenting to a tertiary ocular oncology center over a five-year period who were managed with either external beam radiation or systemic therapy.

## Methods

This study was reviewed by the institutional review board (IRB) at the University of Iowa and was granted an IRB exemption. This study adhered to the tenets of the Declaration of Helsinki. A retrospective review of consecutive patients who were diagnosed with choroidal/ciliary body metastasis by the ocular oncology service at the University of Iowa over a five-year period from 2018 to 2023 was performed. Diagnosis was confirmed by biopsy of the ocular lesion in one case where there was diagnostic uncertainty. Patient age at diagnosis, sex, race, diagnosis of diabetes, primary tumor diagnosis, laterality, treatment modality (targeted biologic, traditional chemotherapy, immunotherapy, hormone therapy, radiation), local response, visual outcome, and systemic outcome were recorded for each patient. Radiation dosimetry was recorded where applicable.

Descriptive statistics were calculated and reported as means. All statistical analyses were performed using Microsoft Excel. A paired t-test was used to compare the mean logMAR visual acuity for patients treated with external beam radiation compared to other modalities.

## Results

Twenty-six patients (33 eyes) with choroidal/ciliary body metastasis were identified. There were 7 right eyes, 12 left eyes, and 7 bilateral cases. Mean age at time of diagnosis of choroidal metastasis was 59 years (18–82). 13 of the patients were female and 13 of patients were male. All patients were Caucasian. Five patients had a diagnosis of type 2 diabetes without retinopathy. Malignancies included lung (8), breast (8), clear cell renal carcinoma (3), esophageal (3), carcinoid tumor (2), squamous cell carcinoma of the tonsil (1), and testicular cancer (1) (**Table 1**). Fourteen patients had a known diagnosis of metastatic cancer at the time of presentation with the choroidal/ciliary body lesion noted either on routine eye exam or due to a complaint of blurred vision. Only one of these patients required diagnostic biopsy of the ocular lesion due to clinical and echographic features overlapping with primary uveal melanoma. Eight patients presented with choroidal lesions clinically consistent with choroidal metastasis that were identified due to a complaint of blurred vision/identified on a routine eye exam and were found to have metastatic cancer on systemic imaging with tissue obtained from non-ocular sites for diagnosis. Four patients presented with choroidal lesions identified on routine exam/due to a complaint of blurred vision in the setting of known primary malignancies that had been in remission, with the ocular disease being the first sign of recurrence.

**Table 1:** Demographic data, visual acuity, and mortality for patients with choroidal metastasis.

**Eye involvement (n=33)**	Right 7
	Left 12
	Bilateral 7
**Gender (# patients)**	13
	13
**Primary malignancy (# patients)**	Lung (8)
	Breast (8)
	Renal cell (3)
	Esophageal (3)
	Carcinoid (2)
	Tonsil (1)
	Testicular (1)
**Treatment (patients n=26)**	Radiation (15)
	Other (11)
**Mean presenting acuity radiation**	0.19 (-0.13-0.88)
**Mean presenting acuity other**	0.32 (-0.13-1.9)
**Mean final acuity radiation**	0.11 (0-3)
**Mean final acuity other**	0.18 (0-0.7)
**Mean time from diagnosis to death**	8 months (1-34)

Fifteen patients (20 eyes) were treated with external beam radiation ranging from a dose of 20-30 Gy (one patient treated at an outside institution with unknown dose). Non-radiotherapy treatments included: targeted biologic therapy; a combination of targeted biologic therapy and hormone therapy; a combination of targeted therapy and immunotherapy; a combination of traditional chemotherapy and immunotherapy; photodynamic therapy (PDT) laser; and immunotherapy alone (**Table 2**). Two patients passed away before treatment was started.

**Table 2:** Specific treatments for patients treated with modalities other than radiation who had follow-up eye exams after treatment.

Tumor type	Traditional Chemotherapy	Checkpoint inhibitor	Immune Therapy	Hormone Therapy	Local Therapy	Response of ocular tumor
Adenocarcinoma lung	–	Osimertinib	–	–	–	Yes
Adenocarcinoma lung	–	–	Pembrolizumab, denosumab, bevacizumab	–	–	Yes
Carcinoid		–	–	–	PDT laser	No
Clear cell renal carcinoma	–	–	–	Ipilimumab/nivolumab followed by nivolumab alone	–	Involuted prior to starting on systemic therapy
Esophageal	Paclitaxel, FOLFOX, FOLFIRI	–	Pembrolizumab, Ramucirumab	–	**-**	Yes
Esophageal	FOLFOX, paclitaxel	–	–	–	–	Yes
Invasive ductal carcinoma of the breast	–	Palbociclib	–	Letrozole	–	Yes
Small cell carcinoma of lung	Carboplatin, etoposide	–	Atezolizumab	–	–	No
Testicular cancer	Bleomycin, etoposide, cisplatin, carboplatin, paclitaxel, isofamide	–	–	–	–	Yes

PDT, photodynamic therapy laser.

Fourteen of 20 eyes treated with radiation had good response defined as decrease in size of the choroidal lesion and improvement of overlying subretinal fluid when present. Of the remaining, six eyes: 1 eye had early response with concern for late recurrence; one eye had a slow response to radiation alone but responded after the addition of immunotherapy after radiation; two eyes had recurrence; and two patients passed away before follow-up after radiation.

For the 13 eyes in patients treated with other modalities who had follow up prior to death (9 patients): one patient had spontaneous involution of the ocular lesion without treatment; one had no response, one had limited response to PDT but very slow, non-visually significant growth of the lesions that were observed; and the remaining patients had a good response to therapy (**Table 1**). Selection of systemic therapy was chosen by the medical oncology service based upon the patient’s systemic tumors, not features of the ocular metastases.

**Figure 1:**
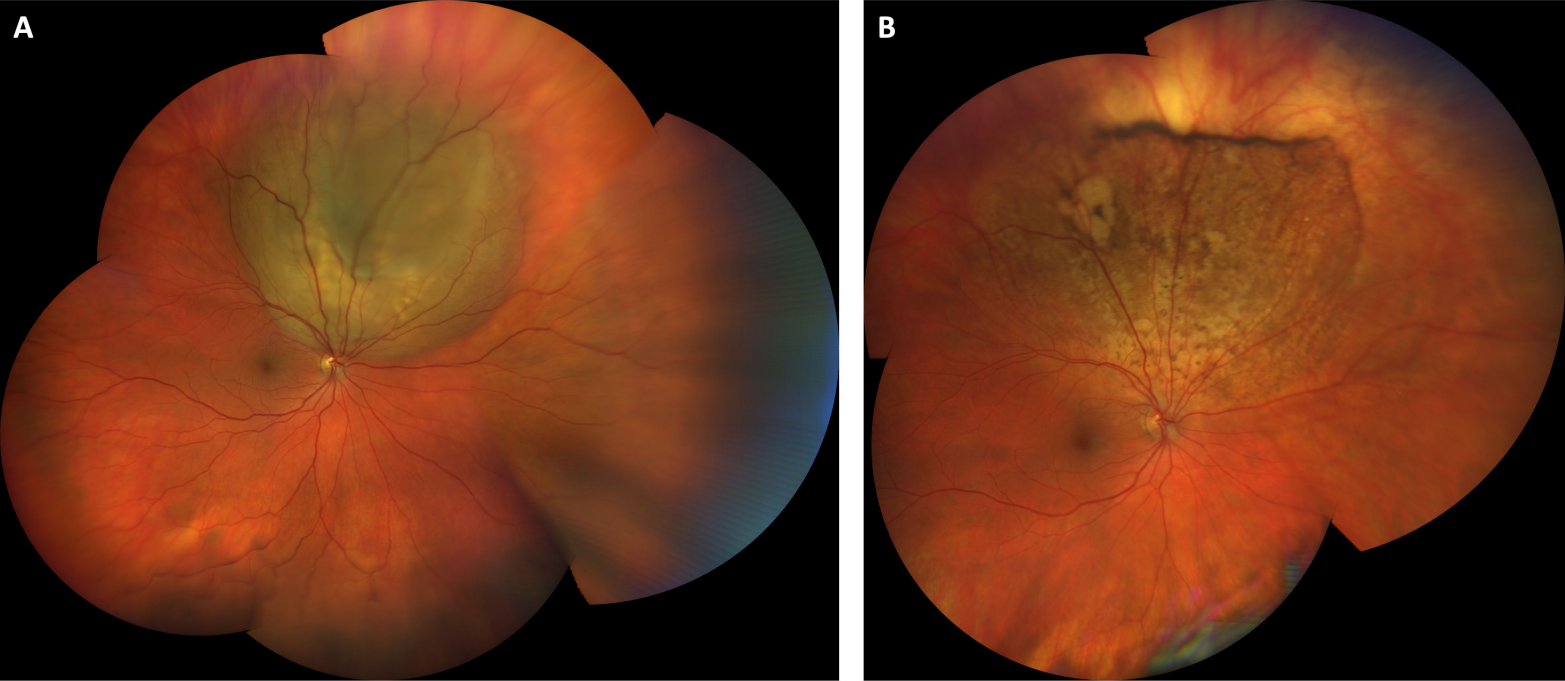
Choroidal metastasis from testicular cancer treated with systemic bleomycin, etoposide, and cisplatin. Wide-field color fundus photograph at presentation shows a temporal, elevated, multilobular amelanotic choroidal metastasis from testicular cancer **(A)**. **(B)** Color fundus photograph one year after initiating treatment with systemic chemotherapy shows an atrophic scar at the site of the prior lesion. Visual acuity was 20/25 at both visits.

**Figure 2:**
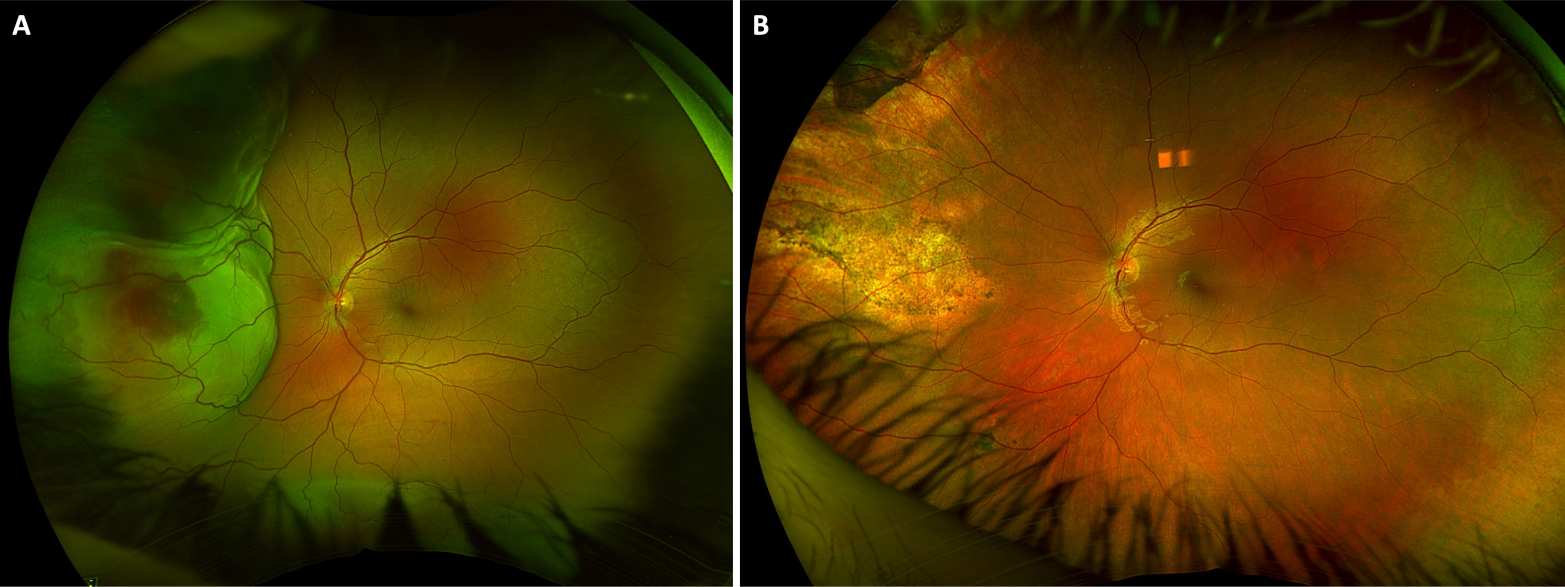
Choroidal metastasis from breast cancer treated with external beam radiation. Color fundus photograph at presentation shows an elevated, amelanotic choroidal metastasis from breast cancer **(A)**. **(B)** Color fundus photograph three months after completion of external beam radiation to the lesion (40 Gy over 20 fractions) shows regression of the lesion to an atrophic scar. Visual acuity was 20/25 at both visits.

The overall average presenting logMAR visual acuity was 0.25 (range -0.13-0.88). The average logMAR presenting visual acuity for patients treated with radiation was 0.19 (range -0.13-0.88) and was 0.32 (range -0.13-1.9) for patients treated with other modalities. The final visual acuity for eyes treated with radiation who had follow up after treatment was 0.11 (range 0-3). Final visual acuity for eyes treated with other modalities was 0.18 (range 0-.70). A paired T-test (Microscoft Excel) showed that this difference was not statistically significant (p=0.48). Fifteen patients were deceased and 10 patients alive at the time of censor. For deceased patients, average time from diagnosis of ocular metastasis to death was 8 months (range 1-34). Illustrative responses to systemic and radiation therapy respectively are shown in **Figures 1**, **2**.

## Discussion

Metastasis to the choroid can occur in almost any systemic malignancy. These tumors can impair quality of life as patients lose vision while battling metastatic disease. Our data demonstrate the real-world challenges in managing these patients as their presentation and clinical course can be extremely heterogenous. Individual histopathologic types of metastatic lesions can behave and respond to therapy very differently and the optimal approach to managing these tumors in the setting of a wide array of new-generation targeted biologic therapies is not known.

While our sample size is relatively small, we found that there was no statistically significant difference in visual outcomes for patients treated with external beam radiation compared to systemic therapy. This is in keeping with other work which has shown success with both approaches in different scenarios (6). Radiation controls many types of choroidal metastasis well, and many patients do not survive long enough after diagnosis to experience visually significant radiation retinopathy (6, 14). However, some types of malignancy such as metastatic germ cell testicular cancer (**Figure 1**) are extremely sensitive to traditional chemotherapy and others have shown good efficacy of systemic targeted therapy particularly for some subtypes of lung cancer (13, 15). The clinical challenge lies in selecting which patients should undergo radiation first line and which patients should receive targeted biologic therapy or traditional chemotherapy in the setting of visually significant disease. We favor a case-by-case, individualized management plan that considers the location and visual threat of the lesion, presence of unilateral or bilateral disease, type of malignancy, presence of mutational status amenable to targeted therapy, ability of patient to travel back for the multiple visits required for treatment, and projected survival time. Patients can experience good visual outcomes with either approach depending upon the clinical scenario.

A particular challenge especially in a more rural setting like our center is travel for repeat treatments with external beam radiation. Other options have included hypofractionated dosing schemes to reduce the number of treatment visits (16). While there is a higher risk for cataract and ocular surface disease with hypofractionated treatment, in patients with limited life expectancy this may be an appropriate option for palliation. Strategies such as lens shielding/lens sparing dosing of radiation can also mitigate this risk (16). External beam radiation carries the risk for dry eye, cataract progression, radiation retinopathy/optic neuropathy, and neovascular glaucoma, but these often appear later and must be balanced with stabilizing vision in the short run for patients with limited life expectancy (16).

Our data are consistent with prior work that shows an overall short time between diagnosis of choroidal metastasis and death (17). However, there is something to be learned from the outlying patients in our series who lived for several years after diagnosis. One of these patients was treated with osimertinib, a tyrosine kinase inhibitor, for metastatic renal cell carcinoma and lived for several years without recurrent ocular disease (18). The durability of response of the ocular tumor and recommendations for ophthalmic surveillance for progression of the ocular tumors while on targeted therapy need to be more clearly defined. Once disease has stabilized, we have adopted an every 3–6-month monitoring approach that coincides with the patient’s systemic imaging evaluations. Careful coordination with the patient’s medical oncologist is important, as progression of ocular lesions may correspond to systemic progression.

## Conclusions

The optimal management of patients with choroidal metastatic tumors in the setting of novel targeted biologic and hormonal therapies is a question that would benefit from a multicenter, prospective study. While these tumors are a sign of end organ involvement from systemic malignancy, they have major implications for patient quality of life and afford a unique opportunity to directly visualize tumor response to systemic therapy. Formalizing strategies to incorporate visual acuity into performance status scales is an area that would benefit from future study as activities such as driving and maintaining reading vision are key aspects of patient quality of life. More work needs to be performed to define the optimal approach to management of these tumors.

## Data Availability

The raw data supporting the conclusions of this article will be made available by the authors, without undue reservation.

## References

[1] Shields CL Shields JA Gross NE Schwartz GP Lally SE. Survey of 520 eyes with uveal metastases. Ophthalmology. (1997) 104:1265–76. doi: 10.1016/S0161-6420(97)30148-1 9261313

[2] Tran T Cypen S Del Valle Estopinal M Tao J. Anaplastic thyroid carcinoma with ocular then orbital metastases. Case Rep Ophthalmol. (2022) 13:76–81. doi: 10.1159/000516053 35350232 PMC8921959

[3] Lokesh AB Kumar R Saheer N Bhatnagar A. Choroid, a unique site for metastasis in Marjolin’s ulcer. J Cancer Res Ther. (2022) 18:1174–6. doi: 10.4103/jcrt.JCRT_617_19 36149181

[4] Qu Z Liu J Zhu L Zhou Q. Clinical features and treatment modalities of rare choroid metastasis from lung Malignancy. Chin Med J (Engl). (2022) 135:1628–30. doi: 10.1097/CM9.0000000000002157 PMC9532045 35838521

[5] Singh A Malik D Singh S Vyas VJ. Choroidal metastasis in pancreatic adenocarcinoma. J Cancer Res Ther. (2022) 18:263–5. doi: 10.4103/jcrt.JCRT_45_20 35381796

[6] Jardel P Sauerwein W Olivier T Bensoussan E Maschi C Lanza F. Management of choroidal metastases. Cancer Treat Rev. (2014) 40:1119–28. doi: 10.1016/j.ctrv.2014.09.006 25451606

[7] Arepalli S Kaliki S Shields CL. Choroidal metastases: origin, features, and therapy. Indian J Ophthalmol. (2015) 63:122–7. doi: 10.4103/0301-4738.154380 PMC4399120 25827542

[8] Liu S Liu X Wang T Zeng C Ren B Yu X. Effective systemic treatment of choroidal metastases NSCLC with surgery after crizotinib: A case report. Front Oncol. (2022) 12:789941. doi: 10.3389/fonc.2022.789941 35433411 PMC9009287

[9] Yamaoka M Igarashi T Shiratori N Miyadera K Sugano T Noro R. A case of binocular metastatic choroidal tumor originating from pulmonary adenocarcinoma successfully treated with molecular target therapy. Case Rep Ophthalmol. (2023) 14:426–32. doi: 10.1159/000530130 PMC10601831 37901630

[10] Matsuyama T Oniwa M Tsuruzono K Yasuda S Yone M Tomioka Y. Improving visual acuity with nivolumab plus ipilimumab plus two cycles of chemotherapy following a diagnosis of lung adenocarcinoma with choroidal metastasis: A case report and literature review. Respirol Case Rep. (2024) 12:e01262. doi: 10.1002/rcr2.v12.1 38045825 PMC10687591

[11] Parakh S Das S Maheshwari S Gupta V Luthra G Luthra S. Regression of choroidal metastasis from breast carcinoma with palbociclib. Int J Retina Vitreous. (2022) 8:54. doi: 10.1186/s40942-022-00398-w 35962417 PMC9373398

[12] Li C Wang XE Yan L Zhao Y Zhu YK. Rapid vision improvement by using icotinib in a patient with bilateral choroidal metastases symmetrically from lung cancer. Clin Respir J. (2023) 17:647–53. doi: 10.1111/crj.13649 PMC10363825 37315930

[13] Maller B Salvatori S Tanvetyanon T. Outcomes of intraocular metastasis from lung cancer in the era of targeted therapy: A systematic review and pooled analysis. Clin Lung Cancer. (2022) 23:e519–e25. doi: 10.1016/j.cllc.2022.07.018 36030188

[14] Niwa M Tomita N Miyakawa A Ayakawa S Takama N Torii A. Clinical outcomes of radiation therapy for choroidal metastases and A literature review. Kurume Med J. (2023) 69:89–97. doi: 10.2739/kurumemedj.MS69120012 37793893

[15] Horwich A Shipley J Huddart R. Testicular germ-cell cancer. Lancet. (2006) 367:754–65. doi: 10.1016/S0140-6736(06)68305-0 16517276

[16] Mathis T Jardel P Loria O Delaunay B Nguyen AM Lanza F. New concepts in the diagnosis and management of choroidal metastases. Prog Retin Eye Res. (2019) 68:144–76. doi: 10.1016/j.preteyeres.2018.09.003 30240895

[17] Shields CL Welch RJ Malik K Acaba-Berrocal LA Selzer EB Newman JH. Uveal metastasis: clinical features and survival outcome of 2214 tumors in 1111 patients based on primary tumor origin. Middle East Afr J Ophthalmol. (2018) 25:81–90. doi: 10.4103/meajo.MEAJO_6_18 30122853 PMC6071342

[18] Rodriguez SM Boldt HC Sullivan HR Rieth JM Zakharia Y Binkley EM. Spontaneous regression of choroidal metastasis from renal cell carcinoma. Am J Ophthalmol Case Rep. (2023) 32:101945. doi: 10.1016/j.ajoc.2023.101945 37886109 PMC10598394

